# Leaf carbon isotope composition: a key proxy for scaling up the best candidates

**DOI:** 10.1093/jxb/eraf007

**Published:** 2025-04-09

**Authors:** Nikolas Souza Mateus, Jose Lavres

**Affiliations:** Center for Nuclear Energy in Agriculture, University of São Paulo, Piracicaba, São Paulo, Brazil; Center for Nuclear Energy in Agriculture, University of São Paulo, Piracicaba, São Paulo, Brazil

**Keywords:** Carbon isotope discrimination, C_4_ photosynthesis, leaf-level water use efficiency, stomatal conductance

## Abstract

This insight article comments on:

**Crawford JD, Twohey RJ, Pathare VS, Studer AJ, Cousins AB.** 2024. Differences in stomatal sensitivity to CO_2_ and light influence variation in water use efficiency and leaf carbon isotope composition in two genotypes of the C_4_ plant *Zea may*s. Journal of Experimental Botany **75**, 6748-6761. https://doi.org/10.1093/jxb/erae286

**The leaf carbon isotope composition (δ^13^C_leaf_) is a highly sensitive and useful tool for assessing differences in water use efficiency (WUE) in major C_3_ crops, as it integrates physiological responses to environmental conditions. However, its use in C_4_ plants remains somewhat ambiguous. In their research, Crawford *et al*. (2025) explore the variations in leaf photosynthetic traits of two *Zea mays* genotypes to environmental changes. The authors offer insights into the mechanisms underlying the speed of stomatal responses to environmental stimuli, which was detectable by δ^13^C_leaf_, minimizing water loss and improving WUE.**

Agriculture is by far the largest consumer of freshwater, utilizing ~70% of global freshwater supplies. As the world’s population is projected to reach nearly 10 billion people by 2050, the demand for food is expected to increase dramatically, intensifying pressure on our already strained freshwater resources. To address this challenge, it is essential to understand how plants will respond to alterations in atmospheric carbon dioxide (CO_2_), precipitation, light intensity, and temperature, among others. Such knowledge is critical for the adoption of sustainable and innovative agricultural strategies that can enhance resilience and productivity in the face of changing environmental conditions, especially in areas with limited resources.

Plants have evolved several strategies to alleviate the impacts of drought stress (reviewed by Ilyas *et al*., 2021). In brief, in the early stages of drought stress, plant responses include physiological adjustments such as stomatal closure to reduce water loss, and regulation of osmotic potential through the accumulation of osmolytes to maintain cell turgor and water uptake, which lead to a decrease in photosynthetic operation (Dewar *et al*., 2022). To withstand extreme and prolonged drought, plants have evolved additional adaptive strategies, such as conduit inactivation or modifications in leaf size (Nadal-Sala *et al*., 2024), key factors influencing carbon dioxide (CO_2_) assimilation, but also water loss through leaf transpiration (*E*). This unavoidable trade-off, often measured as water use efficiency (WUE), serves as a highly integrative indicator of the ability of plants to cope with the challenges posed by climate change (Brodribb *et al*., 2020). Thus, improving WUE through genetic and agronomic advancements has become a critical pathway for ensuring sustainable food production. In simple terms, it means ‘more crop per drop’. Crawford *et al*. (2025) demonstrated that environmental changes triggered substantial variations in net CO₂ assimilation (*A*_net_) and stomatal conductance (*g*_s_), which were detectable by stable carbon isotope composition (δ^13^C_leaf_) and correlated with shifts in WUE in two genotypes of maize. To address this multitiered issue in a wider context, we need to start another section.

## Decoding the multidimensional approaches of water use efficiency

Accurately predicting the variations in leaf photosynthetic traits that regulate WUE is essential for modeling ecosystem-level carbon and water flux. However, measuring WUE is methodologically complex, as it can be determined at different levels, as well as over different temporal scales. At the leaf level and over a short time, it can be described by the intrinsic WUE (WUE_i_), determined by dividing the *A*_net_ per *g*_s_ (Osmond *et al*., 1980) and the instantaneous WUE (WUE_t_), assessed by dividing *A*_net_ per *E* (Farquhar and Richards, 1984). These traits can be obtained through leaf gas exchange measurements, which provide a spatially and temporally restricted snapshot that, while accurate in the short term, is complex to apply across large populations (Hoover *et al*., 2023). Additionally, rapid changes in light, such as those caused by passing clouds, lead to variations in leaf temperature, vapor pressure deficit, as well as physiological responses, thereby affecting WUE_i_ and WUE_t_. At the whole-plant level and over a long time, the long-term WUE (WUE_L_) is determined as the plant dry mass or yield per unit of water used throughout the cultivation (Martin and Thorstenson, 1988). This measure offers a broader perspective when analyzing environmental changes throughout the entire growing season (Santos *et al*., 2024). However, it typically requires continuous measurements over time to accurately assess plant dry mass per unit of water used. This can be labor-intensive and logistically challenging, especially when dealing with large-scale agricultural systems. Moreover, variability in growth conditions and management practices can also be a disadvantage.

It is commonly expected that estimates of the same parameters derived from different methods should be consistent. However, discrepancies often arise, leading to questions about which approach yields the most reliable results. To address these challenges, the stable carbon isotope composition (δ^13^C) has been proposed as a valuable time-integrated proxy for estimating WUE. This approach is particularly useful for modeling dynamic vegetation, as it detects temporal variations in the internal to atmospheric CO_2_ concentration ratio (*C*_i_/*C*_a_), which is driven by *A*_net_ and *g*_s_. In other words, all the impacts on WUE and its implications are fundamentally derived from *A*_net_ and *g*_s_ (Farquhar and Sharkey, 1982).

## Carbon isotope dynamics: a window into plant physiology

**Fig. 1.**
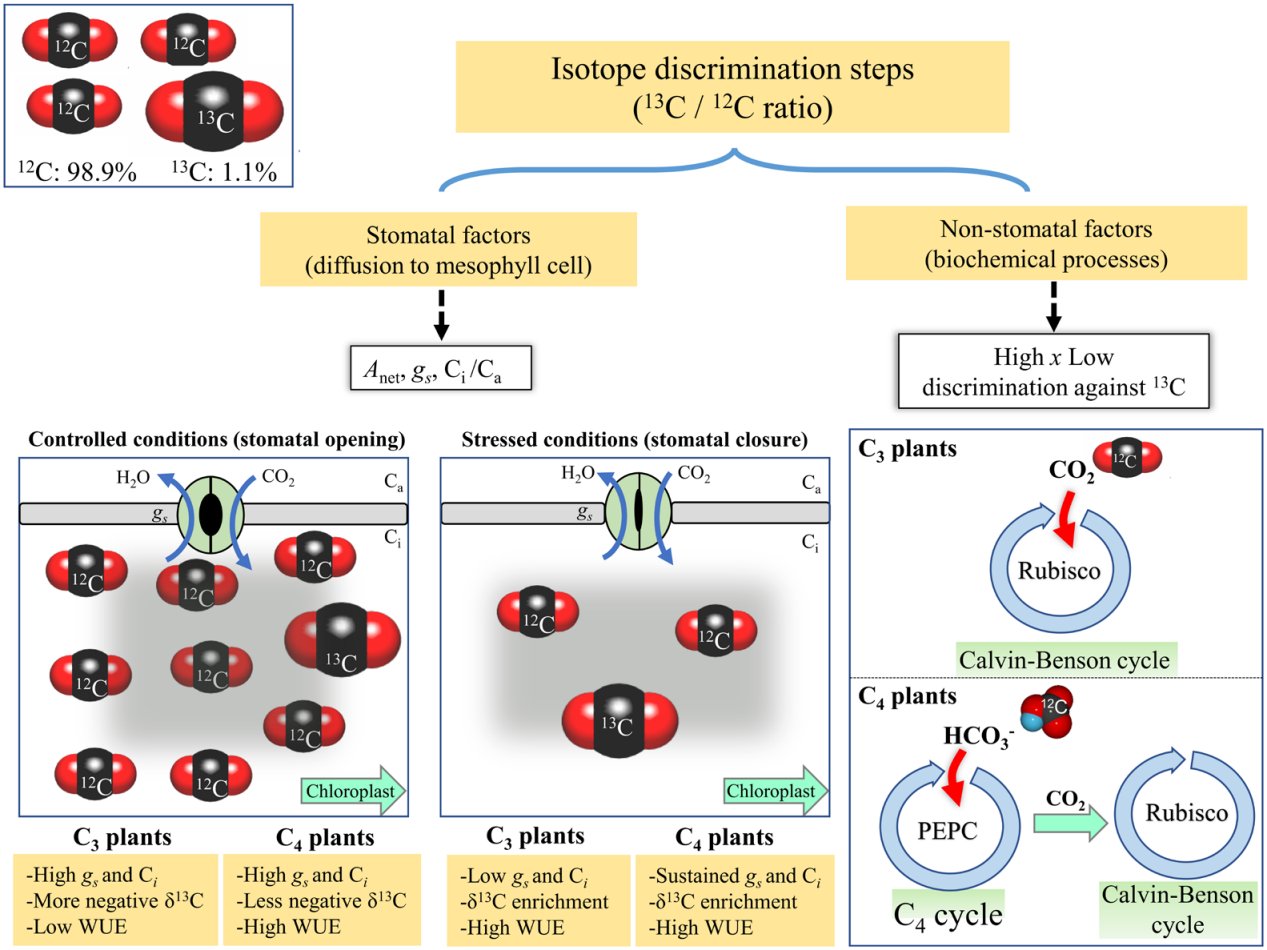
Mechanisms of discrimination against ^13^C during CO_2_ diffusion in C_3_ and C_4_ plants and their relationships with stomatal and non-stomatal processes. Approximately 1.1% of the Earth’s carbon exists as the isotope ^13^C, which contains an extra neutron, making it larger than the more abundant ^12^C. In C_3_ plants, CO_2_ is initially fixed by the enzyme Rubisco, which strongly discriminates against ^13^C, leading to δ^13^C values of –24‰ to –34‰. In contrast, C_4_ plants have a carbon-concentrating mechanism involving the enzyme PEPC before Rubisco activity, which fixes CO_2_ as HCO_3_⁻, resulting in δ^13^C values of –10‰ to –14‰. Under controlled conditions, open stomata maintain high C_i_ while sustaining *A* and WUE, which allows the preferential fixation of ^12^C, thereby reducing δ¹³C values. However, under stressed conditions, stomatal closure reduces *g*_s_ and *C*_i_, leading to the fixation of a higher proportion of ^13^C-enriched CO_2_, thereby increasing δ^13^C. Further details on the physiological and biochemical pathways are provided in the text. Abbreviations: CO_2_ assimilation (*A*_net_), internal (*C*_i_) and atmospheric (*C*_a_) CO_2_ concentration, phosphoenolpyruvate carboxylase (PEPC), stable carbon isotope composition (δ^13^C), and stomatal conductance (*g*_s_).

Understanding carbon isotope composition is vital for predicting plant responses to climate change, as it offers insights into the efficiency of CO_2_ fixation processes and the physiological adaptations that plants undergo during stress conditions. Carbon exists as two stable isotopes: the more abundant ^12^C (98.9%) and the less abundant ^13^C (1.1%) (Fig. 1). Due to the CO_2_ diffusion properties, the heavier ^13^C moves less effectively through the stomatal pores, leading to a 4.4% non-enzymatic discrimination against the ^13^C.

In C_3_ plants, the enzyme Rubisco is responsible for the initial fixation of CO_2_ in the chloroplast, where it discriminates strongly against ^13^C, resulting in a relatively low ^13^C/^12^C ratio and δ^13^C values ranging from –24‰ to –34‰. In contrast, C_4_ plants have an additional carbon-concentrating mechanism upstream of Rubisco. Here, the enzyme phosphoenolpyruvate carboxylase (PEPC) uses bicarbonate (HCO_3_⁻), a hydrated form of CO_2_, as the substrate for the pre-fixation of CO_2_ in mesophyll cells. This pre-fixation step avoids the competition with O_2_ that Rubisco faces in C_3_ plants, while it also creates a concentrated CO_2_ environment around Rubisco in bundle sheath cells. In this context, PEPC is less selective than Rubisco against ^13^C, meaning that C_4_ plants discriminate less against ^13^C and consequently exhibit δ^13^C values ranging from –10‰ to –14‰, reflecting the differences in CO_2_ fixation efficiency between these two distinct photosynthetic pathways (Farquhar and Sharkey, 1982).

In C_3_ plants under controlled growth conditions, when stomata are open and *C*_i_ is high, Rubisco preferentially fixes ^12^C while discriminating against ^13^C, decreasing both WUE and δ^13^C. In the early stages of drought stress, stomatal factors are dominant and the decrease in *g*_s_ leads to a reduction in *C*_i_, causing Rubisco to fix relatively more ^13^C over time (because there is relatively more in the residual pool), which results in ^13^C enrichment, thereby increasing both WUE and δ^13^C. However, during prolonged and severe drought stress, further reductions in *g*_s_, followed by complete stomatal closure, shift the limitation to non-stomatal factors, such as constraints on biochemical processes. At this stage, *C*_i_ may increase due to stomatal dysfunction, ultimately leading to decreased WUE and δ^13^C (Sarabi *et al*., 2019).

In C_4_ plants, the CO_2_-concentrating mechanism maintains a high CO_2_ concentration around Rubisco, minimizing the impact of significant variations in *g*_s_ and *C*_i_. This allows C_4_ plants to sustain *A*, WUE, and δ^13^C compared with C_3_ plants, particularly during the early stages of drought stress.

The study by Crawford *et al*. (2025) represents a huge step forward in our understanding of the stomatal kinetics strategies, for example variations in *g*_s_ rather than *A*_net_, of two closely related maize genotypes to cope with environmental changes, which were found to have consistent and heritable differences in WUE_i_. The authors found that the speed of stomatal responses to environmental stimuli was the primary photosynthetic trait driving WUE. Thereby, the faster stomatal closure, which could be screened by leaf δ^13^C, minimized water loss without compromising *A*_net_, ultimately improving WUE_i_. While numerous studies have primarily indicated anatomical traits as the main factor for stomatal kinetics, such as stomatal density and size, a comprehensive biochemical mechanistic understanding remains incomplete (Lawson and Vialet-Chabrand, 2019). These observations fully align with the results provided by Crawford *et al*. (2025), who found that the quicker stomatal response was likely to be driven by biochemical traits, such as ion transport efficiency, rather than differences in stomatal size alone.

## Moving forward into a thirstier future

With these significant results, the δ^13^C has been validated as a reliable proxy to identify the underlying stomatal traits associated with enhanced WUE in C_4_ crops, which has previously been considered ambiguous. While δ^13^C may be more costly than imaging techniques, its scalability for large-scale screening presents a valuable opportunity rather than instantaneous snapshot measurements. As highlighted by Ozeki *et al*. (2022), the faster stomatal responses observed in C_4_ plants significantly contribute to their higher resource use efficiency relative to C_3_ plants. Thus, engineering major C_3_ crops to match the rapid stomatal response of C_4_ species, coupled with δ^13^C analysis, opens up promising avenues for breeding more water-efficient crop varieties. However, most studies on stomatal kinetics have focused on their consequences, overshadowing a deeper mechanistic understanding of their underlying causes. Given the urgent need for sustainable agricultural practices, further research is essential to uncover the multiple coordinated molecular networks that regulate stomatal kinetics, providing breeders with actionable targets for selecting high-WUE genotypes.

## Data Availability

The data underlying this article will be shared on reasonable request to the corresponding author.
